# A high-density transcript linkage map with 1,845 expressed genes positioned by microarray-based Single Feature Polymorphisms (SFP) in *Eucalyptus*

**DOI:** 10.1186/1471-2164-12-189

**Published:** 2011-04-14

**Authors:** Leandro G Neves, Eva MC Mamani, Acelino C Alfenas, Matias Kirst, Dario Grattapaglia

**Affiliations:** 1 Plant Genetics Laboratory, Embrapa - Recursos Genéticos e Biotecnologia, Parque Estação Biológica, Brasília, 70770-970, DF, Brazil; 2 Graduate Program in Genetics and Breeding, Universidade Federal de Viçosa, 36570-000, MG, Brazil; 3 Department of Cell Biology, Universidade de Brasília-UnB, Brasília, 70910-900, DF, Brazil; 4 School of Forest Resources and Conservation, University of Florida, Gainesville, 32611, FL, USA; 5 Graduate Program in Genomic Sciences and Biotechnology, Universidade Católica de Brasília-SGAN 916 modulo B, Brasília, 70790-160, DF, Brazil; 6 Plant Molecular and Cellular Biology Graduate Program, University of Florida, Gainesville, 32611, FL, USA

## Abstract

**Background:**

Technological advances are progressively increasing the application of genomics to a wider array of economically and ecologically important species. High-density maps enriched for transcribed genes facilitate the discovery of connections between genes and phenotypes. We report the construction of a high-density linkage map of expressed genes for the heterozygous genome of *Eucalyptus *using Single Feature Polymorphism (SFP) markers.

**Results:**

SFP discovery and mapping was achieved using pseudo-testcross screening and selective mapping to simultaneously optimize linkage mapping and microarray costs. SFP genotyping was carried out by hybridizing complementary RNA prepared from 4.5 year-old trees xylem to an SFP array containing 103,000 25-mer oligonucleotide probes representing 20,726 unigenes derived from a modest size expressed sequence tags collection. An SFP-mapping microarray with 43,777 selected candidate SFP probes representing 15,698 genes was subsequently designed and used to genotype SFPs in a larger subset of the segregating population drawn by selective mapping. A total of 1,845 genes were mapped, with 884 of them ordered with high likelihood support on a framework map anchored to 180 microsatellites with average density of 1.2 cM. Using more probes per unigene increased by two-fold the likelihood of detecting segregating SFPs eventually resulting in more genes mapped. *In silico *validation showed that 87% of the SFPs map to the expected location on the 4.5X draft sequence of the *Eucalyptus grandis *genome.

**Conclusions:**

The *Eucalyptus *1,845 gene map is the most highly enriched map for transcriptional information for any forest tree species to date. It represents a major improvement on the number of genes previously positioned on *Eucalyptus *maps and provides an initial glimpse at the gene space for this global tree genome. A general protocol is proposed to build high-density transcript linkage maps in less characterized plant species by SFP genotyping with a concurrent objective of reducing microarray costs. HIgh-density gene-rich maps represent a powerful resource to assist gene discovery endeavors when used in combination with QTL and association mapping and should be especially valuable to assist the assembly of reference genome sequences soon to come for several plant and animal species.

## Background

High-density linkage maps based on transcribed genes are valuable resources to characterize genome structure, gene space distribution and synteny in species for which reference genome sequences are not yet available. Dense gene maps can also support genome sequence assembly [1,2] and the identification of genes underlying loci that control complex phenotypic traits [3]. Most methods used to date for linkage mapping genes in plants such as RFLP, single-strand conformation polymorphism (SSCP), cleaved amplified polymorphic sequence (CAPS) and denaturing gradient gel electrophoresis have very limited throughput [4-6]. High-throughput genotyping of single-nucleotide polymorphism (SNP) derived from genic sequences have allowed large-scale gene mapping. However such efforts require relatively high up-front SNP development, screening and validation costs, which have restricted this technology to the major crop plants [7-9] and a few forest trees [10,11]. Furthermore, current SNP assay methods based on allele-specific primer extension can be of variable robustness in highly heterozygous genomes such as those of forest trees with high nucleotide diversity, frequently above 1% [12].

DNA microarrays have been used to reliably detect genetic differences among individuals [13] as shown initially in the simple genome of yeast [14] and later in *Arabidopsis *by Borevitz *et al. *[15], who termed these genetic variants Single Feature Polymorphisms (SFP). The principle of SFP genotyping is based on the disruption of the hybridization of labeled DNA sequences that are genetically variable relative to a reference sequence used for design of probes on a microarray. Segregating sequence differences among individuals in a mapping population can be detected based on the variation of the hybridization signal [14]. SFPs have been detected by hybridizing total genomic DNA to microarrays in yeast [14], *Arabidopsis *[15,16], and rice [17]. Ronald *et al. *[18], Rostoks *et al. *[19] and Cui *et al. *[20] expanded the scope of the method by hybridizing RNA-derived cDNA or cRNA to expression microarrays in yeast and barley, with the anticipation that polymorphisms in protein-coding DNA sequences that are transcribed into messenger RNA would also weakly hybridize to microarray probes. While this approach reduces the complexity of the pool of sequences hybridized to the microarray, differences in signal intensity that arise as a consequence of sequence variation among genotypes can be confounded with differences in transcript abundance.

SFP mapping involves the detection of probes revealing putative SFP whose behavior as Mendelian markers is then evaluated in segregating populations. SFP have been largely developed for inbred, highly homozygous species. In these studies, a putative SFP is identified based on signal differences between two parental lines. Candidate SFPs are then tested for a bimodal distribution in hybridization signal detected in a segregating population of recombinant inbred lines or backcross progeny, and individuals are assigned to the expected genotypes defined using the signal intensity of the parents as references [21-23]. Using this strategy in *Arabidopsis*, Singer *et al. *[16] built a genetic map with 676 genes by SFPs detection using DNA hybridization while West et al. [21] used complementary RNA (cRNA) hybridization to map 968 genes based on SFP. In the more complex genome of barley, Luo *et al. *[22] reported mapping 1,504 and 1,523 SFPs using cRNA from leaf and embryo from 30 segregating doubled-haploid lines. In the only study to date that attempted to genotype microarray markers in an outcrossing species, Drost *et al. *[24] mapped 324 SFPs segregating 1:1 in a *Populus *pseudo-backcross progeny involving 154 individuals.

In this study we report the detection and linkage mapping of SFPs in a reference pedigree of *Eucalyptus grandis *× *E. urophylla*, two highly heterozygous tree species. Detection of robust SFPs was improved by an initial SFP-discovery step based on the pseudo-testcross strategy followed by using a selective mapping approach to simultaneously optimize linkage mapping and microarray costs. A total of 1,845 genes were successfully mapped, 884 of them positioned with high likelihood support on a framework genetic map. The experimental approach employed is cost efficient and should be readily applicable to large scale mapping of genes in highly heterozygous genomes of several plant species providing a useful resource for downstream applications such as gene discovery, association genetics and assembly of reference genome sequences..

## Results

### Screening for candidate SFPs

**Figure 1:**
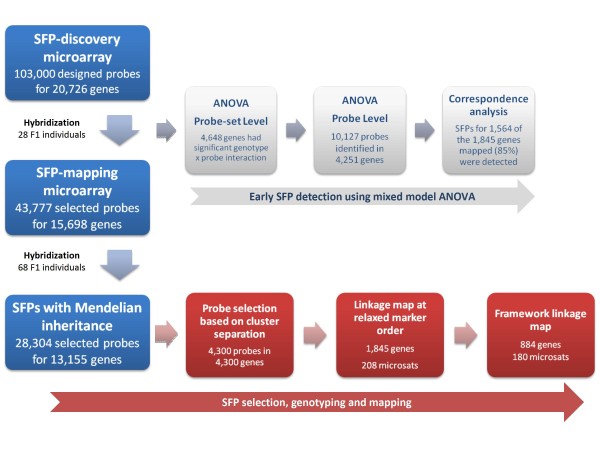
**Flowchart summarizing the steps and outputs of SFP detection and mapping**. The standard procedure involved: (1) probe selection from the SFP-discovery microarray to populate the SFP-mapping microarray and select SFPs with Mendelian inheritance (blue boxes); and (2) downstream data analyses for linkage map construction (red boxes). The mixed-model ANOVA of the SFP-discovery data for an alternative early SFP selection (grey boxes), shows that this approach, had it been taken early on, would have allowed the detection of SFPs sufficient to map 85% of the genes mapped by the standard approach (see text for details).

**Figure 2:**
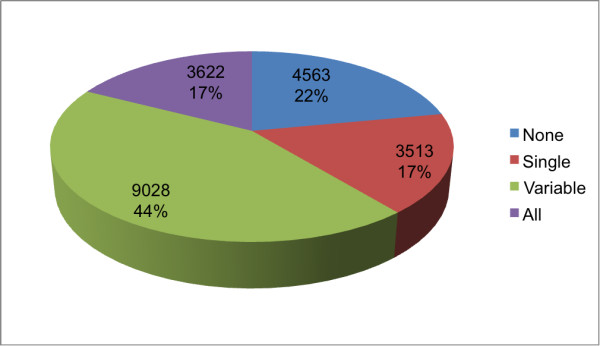
**Distribution of the number of genes for which the probe-sets detected expressed transcripts**. For each gene a probe-set with a variable number of probes (5 or 10) was designed. The number of probes within the probe-set with signal above background was counted and four categories are shown: (None) - when none of the probes displayed signal above background; (Single) - when a single probe in the probe set showed signal above background; (Variable) when more than one but less than the total number of probes in the probe set had signal above background; and (All) when all probes had signal above background.

SFP screening, selection, genotyping and mapping involved a sequence of analytical steps summarized in a flowchart for easy visualization (Figure 1). The SFP-discovery microarray included 103,000 unique probes representing 20,726 unigenes. Analysis of the screening data resulted in a set of 51,661 probes (50% of the total) representing 16,163 genes (78% of the total) detecting expressed transcripts in at least four progeny individuals of the discovery set of 28 individuals. The number of probes detecting transcripts varied considerably across genes (Figure 2) and only for 3,622 genes of the 20,726 assayed (17.5%) all the probes in the probe-set had signal consistently above the negative control threshold in all 28 individuals. Note that in a perfect scenario an expressed transcript would hybridize to all the probes of its corresponding probe set. This indicates that detectable sequence polymorphisms between the short oligonucleotide probes and the hybridized transcripts are the rule rather than the exception.

Following the SFP-discovery step, probes with the lowest mean and standard deviation (measured across the discovery set of individuals) in the signal intensity were removed. Low signal and limited variation in signal intensity in the discovery set was taken as an indication that these probes were less likely to reveal robust SFPs. A new microarray with 43,777 selected probes was designed representing 15,698 genes, with a variable number of probes per probe-set (mean = 2.80 ± 1.41). This new microarray containing selected probes was used to genotype an additional set of 68 individuals drawn from the mapping population using a Selective Mapping approach. This procedure identified the most informative individuals in terms of complementary recombination breakpoints from each parent, optimizing the linkage information to be extracted from these individuals and thus reducing the costs of the SFP genotyping step for map construction.

### SFP genotyping in the mapping population

The averaged, log_2 _transformed, quantile-normalized probe signal detected for each individual was analyzed as described by Drost *et al. *[24] to simultaneously identify and genotype SFPs. Briefly, for each probe the signal intensity measured in each individual offspring was assigned to either one of two distinct clusters using the *k*-means clustering algorithm. Using a chi-square test (α ≤ 0.05), probes showing a 1:1 pseudo-testcross segregation, as well as probes segregating as dominant (3:1) markers were selected. A total of 28,304 probes out of the 43,777 tested followed a Mendelian segregation ratio, with 12,148 segregating 1:1 and 16,156 segregating 3:1 (Table 1), representing a total of 13,155 genes. The degree of separation between the two clusters for a probe was measured by calculating the probability of individuals assigned to one cluster being a member of the other cluster through a modified normal deviate *z_i _*(see Methods). Individuals with *z_i _*equal to or less than 1.96 (*p *≥ 0.05) are likely to overlap with the other cluster and were assigned as missing data to avoid genotype miscalls. When displaying more than 10% missing data, the probe was discarded from further analysis. After this stringent selection step, 5,649 probes representing 4,300 unigenes survived with a slightly larger number of probes segregating in a 3:1 than a 1:1 ratio (3,063 versus 2,586, respectively) (Table 1). For 255 genes, two or more probes revealed SFPs. In those cases, the probe with fewer miscalls and segregating 3:1 was selected over the alternative(s). In spite of their dominant behavior SFP markers segregating in a 3:1 ratio segregate from both parents, provide alellic bridges between the parental maps and when linked in coupling at small recombination distances have essentially the same information content as 1:1 backcross markers. The resulting 4,300 selected SFPs, 1,915 segregating 1:1 and 2,385 segregating 3:1 were used in the subsequent linkage analysis.

**Table 1:** Summary statistics of SFPs selected for linkage mapping using 96 F1 individuals of the *E. urophylla × E. grandis *pedigree

EST collection from which probes were derived*	Total # probes	SFPs selected after χ^2 ^segregation		SFPs selected after z_i _normal deviate		SFPs mapped at low ordering support		SFPs mapped on a framework map	
		1:1	3:1	1:1	3:1	1:1	3:1	1:1	3:1
Contigs	22,598	6,668	8,139	1,407	1,587	570	434	252	271
*E. urophylla*	2,071	567	778	132	134	57	40	20	24
*E. grandis*	10,073	2,445	3,846	496	735	221	134	98	67
*E. globulus*	3,620	1,044	1,361	216	230	93	62	34	29
*E. pellita*	2,133	550	835	144	146	65	36	24	12
Mixed species	3,282	874	1197	191	231	80	53	29	24

Total**	43,777 (15,698)	12,148 (7,764)	16,156 (10,364)	2,586 (2,132)	3,063 (2,583)	1,086	759	457	427

*Unigenes derived from a consensus sequence involving ESTs from different species are indicated as contigs, while singletons are listed by species.

**When several probes were selected per probe set, the number of unique genes represented is shown between parentheses.

### Construction of a gene-rich linkage map for *Eucalyptus*

**Figure 3:**
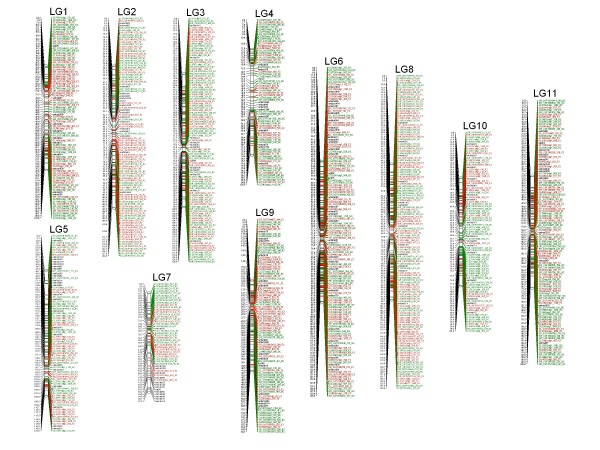
**SFP/microsatellite genetic linkage map of the *E. urophylla *× *E. grandis *pedigree**. EMBRA microsatellites are represented in black, SFPs segregating 3:1 end with the code "F2" while SFPs segregating 1:1 end with code "BC" and are depicted in red and green, respectively. Linkage groups (LG) are numbered following the standardized nomenclature for *Eucalyptus *proposed by Brondani et al. [25].

From the 4,300 SFPs that showed Mendelian segregation, a total of 1,845 SFPs (1,086 segregating 1:1 and 759 segregating 3:1) were successfully grouped at a LOD ≥ 7.0 and could be ordered along the linkage groups together with 208 microsatellites, providing a total map with 2,053 markers with a relaxed marker order support. Details of this full map with all the ordered linkage information of the 1,845 SFPs and 208 microsatellites including map position, original unigene sequence from which the probe was designed, sequence of the probe revealing the SFP, best hit against GenBank non-redundant database and annotation description generated using Blast2GO are presented in a table format (Additional file 1). To provide a gene map with high likelihood support for gene order, a framework map was built using the Round 2 function of Joinmap that removes markers that contribute to unstable order. In the framework map 884 SFPs were mapped together with 180 microsatellites thus resulting in a robust genetic linkage map with 1,064 makers (Figure 3; Additional file 2). Comparative statistics of the full and framework maps is presented (Table 2).

**Table 2:** Descriptive statistics for the 11 linkage groups of the *Eucalyptus *SFP/microsatellite map.

	Full map					Framework map						
Linkage Group	Microsatellites	SFPs		Total # markers	Length (cM)	Microsatellites	SFPs		Total # markers	Length (cM)	Inter-marker distance (cM)	
		All	3:1/1:1				All	3:1/1:1			Mean	SD
1	16	182	66/116	198	100	11	75	37/38	86	102	1.20	1.25
2	26	203	87/116	229	168	17	85	35/50	102	140	1.38	1.55
3	15	154	57/97	169	140	12	95	56/39	107	134	1.26	1.63
4	13	109	42/67	122	107	12	59	38/21	71	103	1.48	1.59
5	23	175	82/93	198	105	23	61	33/28	84	124	1.49	1.76
6	38	233	91/142	271	138	34	104	44/60	138	136	0.99	0.97
7	14	127	66/61	141	93	14	37	22/15	51	98	1.96	1.89
8	13	212	99/113	225	149	12	122	57/65	134	134	1.01	1.16
9	16	143	64/79	159	95	16	75	36/39	91	88	0.98	1.06
10	16	144	42/102	160	111	16	71	47/24	87	108	1.25	1.45
11	18	163	63/100	181	123	13	100	52/48	113	110	0.98	1.55

Average	18.9	167.7	69/98.7	186.6	120.9	16.4	80.4	41.5/38.8	96.7	116.1	1.3	1.4
Total	208	1845	759/1086	2053	1329.4	180	884	457/427	1064	1275	1.21	1.44

Inter-marker distances for the full map is not reported as such estimates might be ambiguous due to the low support for order

From the total number of SFPs mapped in the framework map, 457 segregated in a 1:1 ratio, and 427 in a 3:1 ratio, identified by the acronym BC and F2, respectively. Although no statistical test for clustering was carried out, markers seemed evenly distributed along the linkage groups with no obvious clustering except for some cases on the edge of linkage groups,. Some linkage groups (*e.g. *LG 6 and LG 8) had more genes mapped than others (*e.g. *LG 7) (Table 2). The SFP/microsatellite framework map had an average density of 1.2 cM with 97.5% of the intermarker distances smaller than 5 cM. For only five of the 1,053 intervals the distance was greater than 10 cM, with a maximum of 12.3 cM. Even though the number of mapped markers increased more than five times when compared to the microsatellite-only map, the total genetic length was estimated at 1,275 cM (Table 2) which is within the expected range for *Eucalyptus *[25].

To evaluate the relative contribution of each class of SFP (1:1 and 3:1) to the quality of the genetic map, two separate maps were constructed including microsatellites and either SFPs segregating 1:1 or 3:1. The map that includes only the SFPs segregating 1:1 decreased overall map quality, as illustrated by the expansion of total map length to 1,845 cM based on the generally adopted premise that a shorter map is the most likely hypothesis to explain the data (Table 3). Conversely, the length of the genetic map remained similar (1,130 cM) when only the SFPs segregating 3:1 were included. Thus the detection and use of SFPs segregating 3:1 not only increases the total number of detectable SFPs and consequently mappable genes, but also contributes to a higher quality map possibly due to their role as providers of allelic bridges when linked in coupling.

**Table 3:** Comparative mapping statistics using microsatellites and SFPs with pseudo-testcross (1:1 only) segregation, F2 type (3:1) segregation or both

Linkage Group	Only 1:1		Only 3:1		Both	
	# markers	Length (cM)	# markers	Length (cM)	# markers	Length (cM)
1	45	146	66	92	86	102
2	73	168	70	136	102	140
3	78	208	44	87	107	134
4	59	151	40	67	71	103
5	59	244	63	122	84	124
6	87	197	98	125	138	136
7	39	98	42	109	51	98
8	84	149	81	116	134	134
9	66	138	61	87	91	88
10	73	182	48	88	87	108
11	75	164	66	101	113	110
**Total**	**738**	**1845**	**679**	**1130**	**1064**	**1275**

### Early SFP detection using a mixed-model analysis of variance

The signal intensity data of the 28 individuals of the SFP discovery set was analyzed by a mixed-model ANOVA using the full probe-set for each gene separately to evaluate the correspondence between the putative SFPs detected based on this procedure with those that were linkage mapped by the standard procedure adopted of SFP selection, genotyping and mapping (Figure 1). After correcting the significance threshold of the F tests for multiple testing (false discovery rate < 0.005; *p *< 0.0022), 4,648 genes had significant genotype by probe interaction, corresponding to a putative SFP,. These 4,684 genes, all of them also included in the set of genes represented on the SFP-mapping microarray, encompassed 1,603 of the 1,845 (87%) that were mapped with lower confidence for marker order and 811 of the 884 (92%) that were framework mapped by the standard SFP mapping procedure (Table 4). Specific candidate SFP probes within probe-sets were then searched using the *k-*means clustering analysis and chi square segregation test for Mendelian proportions. A total of 10,127 probes, representing 4,251 genes, were selected in this analysis. This set included probes for 1,564 of the 1,845 genes (85%) that were mapped at a relaxed ordering threshold and 797 of the 884 (90%) that were framework mapped by the standard procedure (Table 4). These results indicate that by testing each probe-set separately for probes that interact with genotypes, instead of 43,777 probes, only 10,127 would have been necessary on the SFP-mapping array to map close to 90% of the genes that were eventually mapped.

**Table 4:** Results of the mixed-model analysis of variance (ANOVA) to identify SFPs.

Description	SFP-detection by conventional linkage mapping	SFP-detection by ANOVA at the probe-set level*	SFP-detection by ANOVA at the probe level*
# probes on SFP-discovery array	103000	103000	103000
# Genes represented on SFP-discovery array	20726	20726	20726
# Genes selected for SFP-mapping step	15698	4648 (100%)	4251 (100%)
# Probes selected for SFP-mapping step	43777	-	10127 (100%)
# Genes mapped (relaxed marker order)	1845	1603 (87%)	1564 (85%)
# Genes mapped (framework marker order)	884	811 (92%)	797 (90%)

Efficiency in detecting mappable SFP at the gene level (probe-set) and probe level by the ANOVA in comparison to the conventional linkage mapping approach

* In parentheses are indicated the percentage of genes or probes in common with those mapped by conventional linkage mapping considered as the benchmark approach

### Impact of the number of probes per probe-set on SFP detection

A total of 762 out of the 16,549 (4.6%) genes for which five probes were used in the probe-set had at least one SFP discovered and were ultimately mapped. In contrast, for the 1,308 genes for which 10 probes were used, the efficiency was almost doubled, with 103 probes eventually mapping, an increase to 7.9% A chi-square test of homogeneity with Yates correction confirmed this difference to be highly significant (χ^2^_d.f. = 1 _= 27.4, *P *< 0.00001) (Table 5).

**Table 5:** Chi-square contingency test for the effect of the number of probes used in the probe-set on the likelihood of mapping the corresponding gene by SFP detection (χ^2^_d.f. = 1 _= 27.4, *P *< 0.00001)

	# probes		
**# Genes**	***5***	***10***	

*Not mapped*	15787	1205	16992
*Mapped*	762 (4.6%)	103 (7.9%)	865*

	16549	1308	

*The total number of mapped SFPs used in the test (865) is lower than the total of SFP mapped (884) because numbers of probes different from 5 or 10 were used for some genes and these were removed from this analysis.

### SFP validation on the *Eucalyptus *draft genome

The linkage relationship of SFPs to microsatellites was validated by using the 4.5X draft genome sequence of *E. grandis*. The location of 64 mapped microsatellites on the supercontigs was confidently determined using WU-BLAST and served to anchor supercontigs to linkage groups. Probes corresponding to 82 of the 884 SFPs were mapped to scaffolds that contained one or more of the 64 anchor mapped microsatellites. The remaining SFP probes mapped to supercontigs, but these were not anchored by any of the 64 microsatellites. Of these 82 SFPs, only 11 were not genetically mapped to the same linkage group as the associated microsatellite, suggesting that they are *trans*-regulated GEMs (Gene Expression Markers). For the remaining 71 SFP's (87%) probes and microsatellites were correctly anchored to the same linkage group located within a 6 cM distance, on average, on the genetic map. Some of these probes could represent *cis*-regulated GEMs instead of SFPs, but mapping of multiple probes within a probe-set to the same genetic position would have been expected in that case. Instead, probe-sets with multiple probes (>2) showing Mendelian segregation were only observed for 66 out of the 884 genes positioned on the framework map.

## Discussion

### Microarray-based SFP genotyping is an accessible approach for gene mapping

In this work, large numbers of SFP displaying Mendelian behavior were readily detected using short (25-mer) oligonucleotide probe arrays in a complex, highly heterozygous plant genome. These SFPs, in turn, allowed the construction of a gene-enriched linkage map providing valuable positional information for hundreds of transcribed genes. The 103,000-probe SFP-discovery oligonucleotide array used in this study was built from a relatively small EST data set for current standards: 88,000 phred-20 filtered 5'-Sanger-sequenced ESTs assembled in 21,428 unique sequences. Currently, much larger and more diverse EST datasets can be rapidly generated at a fraction of the cost using next-generation sequencing technologies as demonstrated for uncharacterized plant genomes including *Eucalyptus *[26,27]. Several millions of ESTs can be generated from pooled samples of RNA for a couple of thousand US dollars providing a very rich source of sequence information to design a similar or much higher density oligonucleotide array. Costs of custom-made high-density microarrays have also dropped significantly and different formats and probe densities are available allowing optimized designs of experiments with multiple samples. By using a two-step SFP discovery and mapping approach combined with selective mapping, one can envisage the construction of a high-density linkage map with thousands of genes for less than 10,000 US dollars. SFP-based gene mapping should therefore prove a very accessible and valuable experimental approach for several plant species.

Recently, a high density Diversity Arrays Technology (DArT) microarray for genome-wide genotyping has been developed for *Eucalyptus *providing over 4,000 polymorphic markers at the population level and an average of 2,211 segregating polymorphic markers per pedigree [28]. When compared to the DArT technology, SFP requires RNA preparation and has a slightly higher cost, while DArT uses DNA and a clever genome complexity reduction method. Nevertheless SFPs have a clear advantage when it comes to specifically mapping genes. The microarray probe design is based on previously known and possibly annotated gene sequences while probes for a DArT array are predetermined and only about 35% of them correspond to coding regions (C. Petroli, unpublished). These two technologies should therefore be considered as highly complementary and distinctively valuable depending on the objectives of the experiment and envisaged downstream applications of the results.

Mapping genes by SFP discovery and linkage analysis would compare well cost-wise to gene mapping by the currently used SNP genotyping platforms, although SNP genotyping costs are rapidly falling. SNP development also requires a transcriptome or sequence database analogous to the one used for SFP probe design. Although targeted single-base assays do require a deeper sequence coverage to adequately select polymorphic SNPs in silico, sequencing costs have dropped drastically so that no major difference in cost between SFP and SNP would happen there. On the other hand, upfront development costs are necessary to screen SNP markers for robustness of the assay and informative polymorphism in a single pedigree. In a recent SNP development study only about 230 out of 768 SNPs (30%) assayed in transcribed genes by the Golden Gate technology segregated and mapped in the exact same mapping population used in this SFP study (D. Grattapaglia submitted). Similar yields of polymorphic segregating SNPs have been found when mapping SNPs in other forest trees without any prior selection of polymorphic SNPs between the parents [10,29]. Given the costs of SNP genotyping on current Illumina chip platforms, the cost of SNP-based mapping the same number of genes mapped by SFPs in this study would be approximately 50% higher. It is important to point out, however, that SFP would not be the method of choice when the same markers have to be assayed repeatedly such as in downstream QTL, association mapping or molecular breeding applications. In such cases optimized SNP platforms would be more adequate. Finally, recent developments in genotyping-by-sequencing methods in plants might soon radically change this scenario by providing orders of magnitude more markers for orders of magnitude lower costs, i.e. tens of thousands of markers for tens of dollars [30,31].

### Pseudo-testcross candidate SFP probe screening increases gene mapping efficiency

SFP discovery and mapping benefited from two key experimental approaches frequently used in linkage mapping studies: pseudo-testcross marker screening [32] and selective mapping [33]. In addition, the flexibility of custom designing oligonucleotide arrays allowed a cost-efficient transition between the SFP-discovery and SFP-mapping steps. Screening for polymorphic SFPs in species for which inbred lines are available have typically relied on comparison of signal intensity between parental lines [21,22,34]. In highly heterozygous species, on the other hand, as SFPs are dominant markers, discovery and mapping SFPs becomes significantly more efficient if parents and a small set of progeny individuals are genotyped to reveal pseudo-testcross segregating markers as originally shown with the RAPD technology [32]. In our experiment the parents were not available. However we used 28 individuals and a cut-off of at least four individuals displaying signal intensity above background to declare a putatively segregating SFP in the SFP-discovery step. Only eight progeny individuals would have sufficed to screen for putatively 1:1 segregating SFP with >99% probability (by the binomial distribution). However by using a larger set of progeny individuals we were also able to target SFPs segregating 3:1 increasing the number of mappable genes and improving the quality of the final linkage map, possibly due to their role as locus bridges and to the fact that most of them were positioned at small distances from other markers therefore providing similar information content for linkage as the 1:1 backcross markers [35]. SFPs segregating 3:1 could potentially be further tested for a 1:2:1 segregation ratio if evidences existed for differential hybridization and detection of the two alleles. Although some SFPs apparently followed such a behavior, we did not attempt to resolve these cases because the putative clusters would be too close to each other therefore resulting in an unacceptable separation statistics and therefore an excessive number of missing data. By genotyping the parents and using them as references for heterozygous genotypes, together with alternative statistical methods, it might be possible to consistently call at least some SFPs in a co-dominant fashion.

Drost et al. [24] pioneered SFPs mapping in a highly heterozygous genomes. They selected candidate SFPs for mapping by comparing signal intensity between the two heterozygous parents of an interspecific *Populus *pedigree using 60-mer oligonucleotide probes. Longer probes were used in an attempt to satisfy a second objective of identifying optimal probes to map GEMs (Gene Expression Markers) and measure gene expression in the progeny. From a total of 384,287 probes designed for 55,793 gene models obtained from the genome sequence, screening resulted in one candidate SFP probe for 12,084 genes. Later, in the mapping phase, only 2,898 SFP probes (0.75%) fitted 1:1 Mendelian expectations and a limited final number of 324 SFPs were mapped. Using only the parents for SFP screening not only ruled out the possibility of selecting SFPs segregating 3:1 but also led to the selection of several candidate SFPs that, in spite of being polymorphic between the two parents, did not segregate in the mapping population. In addition, the use of longer probes, although allowed the identification of 117 GEMs, likely reduced the power to detect SFPs.

In our study we focused exclusively on mapping SFPs over Gene Expression Markers (GEM) and thus used shorter 25-mer probes. An initial set of 103,000 probes for 20,726 genes derived from ESTs was used and 43,777 were selected in the pseudo-testcross SFP-discovery step. This initial screening for SFPs using the progeny, resulted in a much larger proportion of candidate SFP probes (28,304; 27.5%) surviving after testing for Mendelian expectations and 1,845 SFPs were eventually mapped (Table 1). Although comparisons with the *Populus *study are not straightforward due to differences in species, level of polymorphism, gene prediction and especially probe length, these results suggest that screening for SFPs using not only the parents but also the progeny is a better approach to be considered for future SFP mapping experiments.

### The highest density transcript linkage map for a forest tree

To the best of our knowledge our *Eucalyptus *1,845 gene map is the most highly enriched map for transcriptional information for any forest tree species to date. As for the vast majority of plant species, linkage maps for forest trees including *Eucalyptus *have rarely included transcriptional information. Preference has been for more accessible and polymorphic markers such as microsatellites, RAPD, AFLPs and more recently SNPs [36]. In *Populus*, the most gene-dense linkage maps reported had 324 genes mapped by SFPs [24] or 307 unique genes mapped by SNPs [37]. In Oak (*Quercus robur*) 256 transcribed genes were mapped by exploiting microsatellites mined in their sequences [38]. In loblolly pine, a linkage map with 1,635 SNPs representing 1,200 genes was recently published [11], while in white and black spruce (*Picea glauca *and *P. mariana*) 348 genes were mapped using SNP markers [10]. *Eucalyptus *gene mapping effort to date positioned 31 cambium-specific expressed sequence tags (ESTs) and 14 known function genes in a *E. globulus *pedigree using RFLP [39] and eight lignification genes using SSCP [40]. Microsatellites derived from ESTs have been developed for Eucalyptus [41,42] and over 100 of them have been recently mapped (D. Faria, unpublished) although throughput is clearly unacceptable to map large numbers of genes. In our study, SFP detection and mapping was done on a segregating population derived from an interspecific cross between two elite genotypes of *E. urophylla *and *E. grandis*. Both species are highly heterozygous as revealed by microsatellite surveys [25]. High levels of nucleotide sequence diversity have been reported for *E. grandis *(~1%) [26] and even higher when a larger sampling was carried out in *E. camaldulensis *(3-5%) [43]. We predict that equivalent SFP and gene mapping efficiencies will be attainable in other *Eucalyptus *species and pedigrees given the generally high levels of nucleotide diversity seen in species of this genus. Genes revealing SPFs are likely to be more variable at the DNA sequence level. Perhaps these genes are less constrained by selection or, in the present case, display a higher level of inter specific differentiation given the hybrid nature of the cross. Although speculative at this point, there may be some evolutionary significance associated with the detection of SFPs and consequently with the genes mapped by this method. Gene-rich linkage maps of SFP markers can therefore provide an excellent framework to carry out genus wide high-density comparative mapping in *Eucalyptus *and related genera in the Myrtaceae and possibly derive some appealing evolutionary hypotheses.

The 1,845 transcribed gene map reported in this study represents a significant improvement beyond the number of genes previously positioned on *Eucalyptus *genetic maps. To generate preliminary functional annotation of the 1,845 genes mapped, we employed Gene Ontology (GO) mapping using Blast2GO (B2G) [44]. This automated tool designed for non-model species extracts and maps GO terms to the sequences to generate candidate annotations. This was done using a locally installed instance of B2G using the output of a blastx against GeneBank (e-20) as starting point. A total of 1,068 unigenes had a significant blast hit and, from these, B2G annotated GO terms to 984 genes and 868 genes had annotation ultimately mapped after applying B2G default filters. Details of this annotation for both the full and framework maps are available in Additional files 1 and 2, respectively. An improved annotation of these mapped genes will have to wait for the annotated version of the *Eucalyptus grandis *genome.

Nevertheless, this gene map not only offers a coarse first glance at the still undefined gene space in *Eucalyptus*, but also provides a useful resource for improving the assembly of the *E. grandis *genome, currently in progress [45,46]. In poplar, even a relatively modest 324 SFP map allowed localizing over 35 million base pairs of previously unplaced whole-genome shotgun (WGS) scaffold sequence to putative locations in the genome of *Populus trichocarpa *[24]. Out of the 1,845 genes successfully mapped, 884 of them were positioned with high likelihood support for gene order (Table 2). Robust marker ordering for all 1,845 genes, if desired, could be obtained by increasing the number of progeny individuals by selective mapping. The 961 genes (1,845-884 = 961) mapped with a relaxed order support are somewhat equivalent to bin mapped markers, i.e. markers that are assigned to specific map segments (bins) typically using less than 20 individuals and loosely assigning them to map bins [33,47-50]. A much higher mapping precision than bin-mapping was attained in our study, however, by using 68 individuals chosen by selective mapping plus the 28 used in the SFP screening step. Possibly several hundreds more SFPs and corresponding genes that were left out in the linkage and ordering analysis could be assigned to linkage groups by using such a coarse bin-mapping approach to provide approximate positions. Bin-mapping using only 20 individuals or less would be a very appealing approach to map large numbers of genes by SFP genotyping in the vast majority of species that suffer from limited resources providing an initial sense of the gene space and candidate positional genes for further genomics undertakings.

### SFP detection and mapping is improved by using larger probe-sets and ANOVA based SFP-discovery

Our results showed that two procedures improve the overall efficiency of SFP detection and mapping. The first one is to use a mixed-model ANOVA on the raw signal intensity data of the SFP discovery set data to analyze the full probe-set for each gene in a separate model. A retrospective analysis of the discovery set data showed that this approach allows early detection of high quality SFPs and, thus, could reduce experimental costs by optimizing the selection of probes to populate the final SFP-mapping array (Figure 1). Had we used this additional analytical procedure on the discovery set data early on, we would have successfully detected informative probe-sets and specific probes within probe-sets for the vast majority of the genes that were eventually mapped by conventional linkage mapping. Instead of using probe-sets for 15,698 genes in the SFP-mapping array, only 4,684 genes that showed a significant genotype by probe-set interaction would have been represented on the array. Furthermore, instead of 43,777 probes, only 10,127 would have been used on the final SFP-mapping array, significantly reducing overall experimental costs while keeping a very similar final gene mapping efficiency (Table 4).

The second procedure that improves SFP detection and mapping is to use a large number of probes per probe-set. On average, five unique non-overlapping 25-mer probes were designed and used to represent each unigene on the microarray. However, for a subset of 1,308 genes a probe-set of 10 probes was used to assess the impact of using a larger number of probes on the likelihood of detecting a mappable SFP. Considering a similar mutation rate along the coding region, we anticipated that doubling the number of screened probes would result in an equally proportional increase in the detection of probes with SFPs. An almost two-fold increase from 4.6% to 7.9% was observed and highly significant (Table 5). Using more probes per unigene does result in a significantly increased likelihood of discovering a segregating SFP that will allow mapping the corresponding gene. This result is relevant when considering mapping specific genes using SFP markers. In such a scenario, the recommendation would be to design the largest possible number of non-overlapping probes so as to scan the whole extension of the target gene for detectable sequence polymorphisms. This approach would be particularly useful to map a large number of putative candidate genes in an attempt to co-localize such genes and mapped QTLs for species that still lack a reference genome sequence.

### A protocol to optimize gene mapping by SFP genotyping

Results of our study allow us to propose an experimental procedure to build high-density transcript linkage maps in less characterized plant species with no reference genome yet available with a concurrent objective of reducing microarray synthesis and hybridization costs: (1) a large EST collection of several tens of millions of reads is quickly constructed at a relatively low cost by pooling and sequencing RNA samples from several different individuals and tissues using next generation sequencing technologies; (2) a high-density microarray with large numbers (10 or more) of probes for each one of all available unique genes identified in the EST database is designed to be used as a SFP-discovery array; if particular genes are targeted for mapping, the number of probes designed for them should be maximized; (3) RNA from the two parents and sixteen offspring individuals of a mapping population is hybridized to this SFP-discovery array; candidate SFP probes within probe-sets interacting with genotypes, representing genes with a significant expression signal difference between the parents and/or segregating in the progeny are identified by a combined ANOVA and clustering analysis (both segregation ratios - 1:1 or 3:1 - can be initially detected with 16 individuals with a probability > 99%); selected probes are then used to populate a SFP-mapping array; (4) RNA from a larger progeny set of a few tens of individuals (depending on resource availability), drawn from a larger mapping population using selective mapping is used for final SFP genotyping, segregation analysis and linkage mapping using the SFP-mapping array. The efficiency of this protocol can be enhanced by carrying out the above steps in more than one pedigree simultaneously, thus increasing the probability of detecting segregating SFPs for a larger number of genes and eventually mapping them.

## Conclusions

Our study shows that starting from a relatively modest EST resource, SFP discovery and mapping using high-density oligonucleotide arrays is a powerful approach to quickly position several hundreds or thousands of genes on a reference map for any organism. Using this experimental approach in *Eucalyptus *we generated the most highly enriched map for transcriptional information for any forest tree species to date with 1,845 genes. The large number of genes mapped by SFP detection provides useful information for several genomic applications and offers a preliminary glimpse at the *Eucalyptus *gene space. Gene-rich maps represent a useful resource for comparative genomics and gene discovery for plant and animal species for which reference genomes are still to come. When used in combination with QTL and association mapping data, the co-localization of mapped genes with high resolution QTL mapping data could rapidly lead to positional candidate genes for which specific SNPs could be developed and used in association genetics studies. Additionally, gene mapping information could increase the relative weight of specific gene-rich regions in predictive Genomic Selection models to assist forest tree breeding programs for multiple complex quantitative traits [51]. Finally, gene-rich high-density linkage maps will be especially valuable when time comes to assemble reference genome sequences, a likely task for several plant and animal species in the very near future.

## Methods

### *Eucalyptus *pedigree and microsatellite map

A mapping population of 188 F_1 _individuals derived from an interspecific cross between *E. urophylla *(genotype U15) and *E. grandis *(genotype G38) planted in a single-tree plot design in the municipality of Guaiba, Brazil (30° 06' 50" S 51° 19' 30" W) was genotyped at 180 microsatellites markers following procedures described elsewhere [52]. An integrated genetic linkage map was generated that followed the marker ordering of the reference map by Brondani et al. [25] with a few additional mapped markers recently described [41,42]. For 28 randomly selected progeny individuals, biological replicates in the form of clonal ramets were available and were used in an initial SFP-discovery step. To optimize subsequent linkage map construction, while keeping microarray synthesis and hybridization cost to a minimum, 68 individuals were selected for SFP genotyping and mapping analysis out of the 188 available, based on the distribution of recombination breakpoints inferred from the microsatellite data set using a selective mapping (also called bin mapping) approach implemented in MapPop 1.0 [53]. Selective mapping was carried out for each parental map separately. From the 68 individuals selected from each parental map data, 41 overlapped between the two selected data sets and the others were selected among the remaining individuals to equally represent each parental map.

### Tissue collection, RNA preparation, labeling and microarray hybridization

For the 96 individuals of the segregating population used for SFP mapping, differentiating xylem was collected from actively growing trees at age 4.5 years grown in a common field. Xylem was chosen as it typically holds a wider transcript diversity when compared to other common tissues such as leaves or flowers (G. Pasquali, unpublished), thus potentially increasing the pool of genes sampled. An area of approximately 20 × 10 cm had the bark removed and xylem samples were scraped. Immediately upon collection, tissues were stored in dry ice prior to being lyophilized. Total RNA extracted [54] was treated with RQ1 RNase-free DNase (Promega, Madison, WI, USA), purified in mini-spin columns (RNeasy Plant Mini Kit, Qiagen, Valencia, USA) and the quality was evaluated on agarose gels and for adequate 260/280 nm and 230/260 nm absorbance ratios on a Nanodrop 2000 (Thermo Fisher Scientific, Waltham, MA, USA). The Two-Color Quick Amp Labeling Kit (Agilent Technologies, Santa Clara, CA, USA) was used to synthesize complementary RNA (cRNA) following the manufacturer's protocol, except that reagent volumes were reduced to one-half. All samples yielded enough labeled cRNA for hybridization as recommended by the manufacturer. Samples were hybridized at the Interdisciplinary Center for Biotechnology Research (ICBR) of the University of Florida following Agilent's protocol, except for lowering the hybridization temperature to 55°C. The sequence information for all probes used in the SFP-discovery array is presented (Additional file 3). Expression data for both arrays and all genotypes were deposited in the Gene Expression Omnibus with the superseries GSE24196.

### SFP-discovery microarray experimental design and analysis

A large format microarray was designed with the objective to screen a large set of oligonucleotide probes using a subset of 28 individuals (referred hereafter as the *discovery *set) of the mapping population and to select probes revealing putatively mappable SFPs. These 28 individuals were all progeny individuals and had been clonally propagated to provide biological replication for the microarray analysis. The parents of the mapping population were not available and in fact were not necessary for the pseudo-testcross mapping approach. A loop design [55] was adopted in the SFP-discovery experiment using the discovery set with two biological replicates. Probes were developed based on an expressed sequence tag (EST)-derived unigene set from four *Eucalyptus *species (*E. grandis, E. urophylla, E. globulus, E. pellita*) during the Genolyptus project [56]. A probe-set of ten non-overlapping 25-mer oligonucleotides were initially designed for each of 21,428 unique sequences using the eArray software package (Agilent Technologies). From the 214,218 designed probes, five probes with the most consistent GC content and melting temperature, as well as low probability of cross-hybridization, were selected to represent each unigene. For 2,868 unigenes, fewer than five high-quality probes could be designed, and were all included in the microarray. In addition, ten probes were included for a random subset of 1,308 unigenes. In total the SFP-discovery microarray comprised 103,000 probes representing 20,726 unigenes with a variable number of non-overlapping probes per gene, the majority of them (16,549) represented by five probes. The complete list and detailed information for the probes used in this *Eucalyptus *SFP-discovery microarray is supplied (Additional file 3). Short oligonucleotide probes (25-mer) were used because they are more sensitive to single base mutations or indels than longer probes [57]. Twenty-six negative control probes used in previous studies [1,24] were also included in the microarray. Signal data was processed by log2 transformation, quantile-normalization using the Affy package on R [58] and averaging the signal from the same genotype. Any individual probe was considered not to be representing an expressed mRNA when the signal was below 0.297 relative fluorescence units, which represented 90% of the signals for the 26 negative control probes present on the array. When 25 or more individuals (90%) had their signals below that threshold, the probe was considered not to have a corresponding mRNA expressed. This resulted in 51,661 probes for 16,164 genes. To populate the 44 K genotyping-array we reduced the number of probes by removing those with the lowest mean and standard deviation, resulting in 43,777 probes for 15,698 genes.

### SFP-mapping experimental design and analysis

The microarray used for the full scale progeny genotyping included 43,777 probes representing 15,698 genes selected after the SFP-discovery step, together with 26 negative control probes. A total of (68) F1 progeny individuals were genotyped using the SFP-mapping microarray and data for 28 individuals from the discovery set was retrieved for the same 43,777 probes, totaling 96 individuals. The raw median signal intensity for each probe of all 192 hybridizations (two biological or technical replication per genotype) were log_2 _transformed and quantile-normalized. The averaged normalized data for each of the 96 individuals was used for the simultaneous identification and genotyping of putative SFP using a *k*-means clustering method modified by Drost *et al. *[24] from Luo *et al. *[22]. Briefly, the learning algorithm allocates the signal intensity of each genotype into two distinct clusters on a per-probe basis [24]. Probes segregating in Mendelian ratios (1:1 or 3:1) were identified based on a goodness-of-fit chi-square test at α ≤ 0.05 and those that did not follow this expectation were excluded from further analysis. Finally, based on the mean and standard deviation calculated for each cluster, the probability that the individual assigned to one cluster is not a member of the other was calculated using the modified normal deviate *z_i _*= (*x_i _*- *m_j_*)/*s_j_*, where *x_i _*is the signal intensity of an individual from cluster *i *and *m_j _*and *s_j _*are the mean and standard deviation of the cluster *j *[22]. Individuals with *z_i _*≤ 1.96 have a probability equal to or greater than 5% of belonging to the alternative cluster and, therefore, a greater chance of being ambiguously genotyped. Only probes for which there was less than 10% of missing data were selected for analysis in the full progeny set. Selected probes segregating 1:1 (pseudo-testcross) and 3:1 (dominant markers in F2) received the acronym of BC and F2 reflecting their segregation configurations. In some instances, more than one probe within a probe-set was selected as a candidate SFP for genotyping the mapping population. In those cases, an empirical iterative method was developed to select the best possible probe for the probe-set as follows: (i) the probe with less missing data was selected; (ii) probes revealing F2 segregating SFPs were preferentially selected over BC; and (iii) probes with the greatest gap between cluster means were selected. Clustering analyses were implemented on SAS 9.1 using *Proc Fastclus *and filtering steps were implemented on JMP 7.0 (SAS Institute, Cary, NC, USA).

### Genetic map construction

SFP genotyping information for the 96 individuals was integrated with 180 microsatellites. JoinMap 3.0 [59] was used to construct an integrated map using the following parameters: population type CP; grouping at LOD> 7; recombination fraction ≤ 0.4; ripple value = 1; jump in goodness-of-fit threshold (the normalized difference in goodness-of-fit chi-square before and after adding a locus) = 5; Kosambi mapping function. Marker ordering with Joinmap was carried out by simulated annealing. A complete map with all mapped SFPs was generated using the Round 3 function of Joinmap that fits all grouped markers ignoring the requirements of maximum allowed reduction in goodness-of-fit and therefore has a relaxed marker order support. A map with high likelihood support for gene order, a framework map was built using the Round 2 function of Joinmap that removes markers that contribute to unstable order. Microsatellites anchoring the SFP markers had been previously mapped using both MapMaker and Joinmap and thus provided a reference framework map ordering on which the SFP markers could be robustly mapped.

### SFP-detection using a mixed-model analysis of variance on the discovery set

From the SFP-discovery microarray, log_2 _transformed, quantile-normalized data for the 20,726 unigenes with expression profiled in 28 biologically replicated individuals were analyzed using a mixed-model analysis of variance (ANOVA) as outlined by Wolfinger *et al. *[60] and Rostoks *et al. *[19]. The linear model is:

Where, y_gjk _is the log2, quantile-normalized measurement from the *g*^th ^gene (*g *= 1,..., 20726), *j*^th ^array (*j *= 1,..., 28), and *k*^th ^dye (*k *= 1, 2); μ is the overall experimental mean; *A *is the global main effect for microarrays; *D *is the global main effect for dies, *AD *is the global interaction effect of microarrays and dies, and ε is the residual. Dye was considered a fixed effect whereas microarray and interactions were considered random effects. The residuals from this model (referred as r_gipj_), generated for each probe and genotype, were used as input for a second gene-specific linear model including genotype, probe and genotype by probe-set effects as sources of variation. The model was:

With *G *being the effect of genotype (*i *= 1,..., 28), *P *being the effect of probes (*P *= varies according to gene from 1 to 10), *GP *being the interaction effect of genotypes and probes, and *y *being the random error. Genotype, probe and their interaction were considered as fixed effects, microarray as random effect, and mean and error respectively as fixed and random effects. This model was fit gene-by-gene and, thus, all values were indexed at the gene level. The ANOVAs were implemented in SAS 9.1 (SAS Institute, Cary, NC, USA). F-tests for the genotype by probe interaction effect of each gene were performed and the probabilities were corrected for multiple testing using a modified false-discovery rate [61]. Significance of this test identified genes in which probes within probe-sets behaved differently among genotypes, therefore indicating a candidate segregating SFPs.

### *In silico *validation of SFPs on the *Eucalyptus grandis *draft genome

To validate the linkage relationship of SFPs to microsatellites and verify the occurrence of true SFP as opposed to GEMs (Gene Expression Markers) for the mapped genes, we first located a set of microsatellites with high confidence to the unordered supercontigs of the 4.5X draft of the *Eucalyptus grandis *genome (available at http://eucalyptusdb.bi.up.ac.za) using WU-BLAST [62] with an e-value threshold of e-20. This analysis allowed anchoring sets of supercontigs to linkage groups. Subsequently all probes for the 884 SFPs were tentatively mapped to the supercontigs again using WU-BLAST and same e-value threshold. Validation was based on the observation of the proportion of SFPs that could be mapped to the same supercontig as the microsatellites. SFPs that did not map to the same linkage group were interpreted as being GEMs.

## Authors' contributions

LGN designed the microarray, collected xylem samples and prepared RNA, genotyped the population, analyzed the data, summarized the results and wrote the first version of the manuscript. EMCM constructed the microsatellite linkage map. ACA provided logistic support for sample collection and RNA extractions. MK helped supervise and design the experiments and assisted in data analysis. DG conceived and supervised the study, carried out initial SFP detection experiments, edited and complemented the final version of the manuscript. All authors read and approved the final manuscript.

## Supplementary Material

Additional file 1 **Full map with 1,845 SFPs and 208 microsatellites**. Ordered linkage mapping data for the full map built with JoinMap at relaxed ordering threshold involving 1,845 SFPs and 208 microsatellites. Information includes the Unigene ID in the original EST database, Probe_ID, SFP Marker ID, Linkage group, Position in centiMorgan, Probe_sequence, Unigene_sequence, number of probes designed in the probe set, Best BLAST hit against nr, Identity and e-value, annotation description generated using Blast2GO, number of blast hits with e-value equal or greater than -20, mean similarity for these hits, number of GO terms mapped, GOs that were annotated to the gene and enzyme codes generated by the annotation. Click here for file

Additional file 2 **Framework map with 884 SFPs and 180 microsatellites**. Ordered linkage mapping data for the framework map built with JoinMap at high likelhood support for ordering involving 884 SFPs and 180 microsatellites. Information includes the Unigene ID in the original EST database, Probe_ID, SFP Marker ID, Linkage group, Position in centiMorgan, Probe_sequence, Unigene_sequence, number of probes designed in the probe set, Best BLAST hit against nr, Identity and e-value, annotation description generated using Blast2GO, number of blast hits with e-value equal or greater than -20, mean similarity for these hits, number of GO terms mapped, GOs that were annotated to the gene and enzyme codes generated by the annotation. Click here for file

Additional file 3 **Eucalyptus SFP screening 103k-probe array**. List of the 103,000 25-mer oligonucleotide probes designed from 20,726 *Eucalyptus *unigenes and used in the SFP-discovery microarray. Click here for file
